# Model-Based Evaluation of Spontaneous Tumor Regression in Pilocytic Astrocytoma

**DOI:** 10.1371/journal.pcbi.1004662

**Published:** 2015-12-10

**Authors:** Thomas Buder, Andreas Deutsch, Barbara Klink, Anja Voss-Böhme

**Affiliations:** 1 Zentrum für Informationsdienste und Hochleistungsrechnen (ZIH), Technische Universität Dresden, Dresden, Germany; 2 Fakultät Informatik / Mathematik, Hochschule für Technik und Wirtschaft Dresden, Dresden, Germany; 3 Institut für Klinische Genetik, Medizinische Fakultät Carl Gustav Carus, Technische Universität Dresden, Dresden, Germany; University of California, Irvine, UNITED STATES

## Abstract

Pilocytic astrocytoma (PA) is the most common brain tumor in children. This tumor is usually benign and has a good prognosis. Total resection is the treatment of choice and will cure the majority of patients. However, often only partial resection is possible due to the location of the tumor. In that case, spontaneous regression, regrowth, or progression to a more aggressive form have been observed. The dependency between the residual tumor size and spontaneous regression is not understood yet. Therefore, the prognosis is largely unpredictable and there is controversy regarding the management of patients for whom complete resection cannot be achieved. Strategies span from pure observation (wait and see) to combinations of surgery, adjuvant chemotherapy, and radiotherapy. Here, we introduce a mathematical model to investigate the growth and progression behavior of PA. In particular, we propose a Markov chain model incorporating cell proliferation and death as well as mutations. Our model analysis shows that the tumor behavior after partial resection is essentially determined by a risk coefficient *γ*, which can be deduced from epidemiological data about PA. Our results quantitatively predict the regression probability of a partially resected benign PA given the residual tumor size and lead to the hypothesis that this dependency is linear, implying that removing any amount of tumor mass will improve prognosis. This finding stands in contrast to diffuse malignant glioma where an extent of resection threshold has been experimentally shown, below which no benefit for survival is expected. These results have important implications for future therapeutic studies in PA that should include residual tumor volume as a prognostic factor.

## Author Summary

The most common brain tumor in children and young adults is pilocytic astrocytoma (PA). This tumor is usually benign and often follows an indolent course. The treatment of choice is resection and the prognosis is very favorable if total excision can be achieved. However, due to the location of the tumor, only partial resection is possible in many cases. Partially resected PA could spontaneously regress, regrow or even progress to a more aggressive type of PA. We develop a mathematical model which describes the growth, progression and regression of PA. We are able to quantitatively predict the chance for regression in dependency of the remaining tumor size. This prediction has the potential to provide decision support to clinicians after partial resection of benign PA. Furthermore, our results imply that there is no resection threshold for PA below which no survival advantage is provided. This finding stands in contrast to malignant brain tumors where such a threshold has been experimentally shown.

## Introduction

Pilocytic astrocytoma (PA) is the most common pediatric brain tumor and the second most frequent tumor in childhood [1]. Three of four cases are diagnosed up to an age of 20 years with the highest age incidence between 5 and 15 years. PA is usually benign, often follows an indolent course and is mostly slow-growing [2]. In children, PA most frequently occurs in the cerebellum but can develop in the entire neuroaxis. Surgery is the treatment of choice [3]. If total excision is achieved, the prognosis is favorable with more than 90% of patients being cured [4]. However, in many cases tumor location in critical or deep areas (such as brain stem, optic pathway, or hypothalamus) restricts resection options and alternative management options are required [5, 6]. Patients with only partial resection have a worse and highly unpredictable prognosis [4, 5]. Tumors can regrow or even progress to a more aggressive tumor [3, 7–11] but spontaneous tumor regression of PA has also been observed [4, 12–15] and is a common phenomenon. A recent review in [14] estimates a fraction of 14% of all residual cerebellar astrocytoma that regress spontaneously. Other studies claim an even higher portion [16]. While regression of PA after partial resection is reported in many case series [12–16], the influence of the residual tumor size has not been evaluated yet. Moreover, the management for patients in whom complete resection cannot be achieved is still unclear. Due to the chance of regression and the indolent nature of PA, some authors propose a wait and see strategy in order to avoid potential risks induced by further therapies [4, 7, 14]. Other authors favor an aggressive surgical resection in combination with additional treatment strategies, like radiation and chemotherapy to control tumor growth [15, 17, 18].

On the molecular level, it has been shown that activation of the mitogen-activated protein kinase (MAPK) pathway is sufficient to induce the development of PA. This leads to the hypothesis that PA is a single-pathway disease [19, 20]. Furthermore, PA usually harbor only one alteration within the MAPK pathway. The majority of mutations are activating changes in the BRAF gene, the most common is the KIAA1549-BRAF fusion, but also other activating mutations have been described. A more aggressive behavior of PA is observed if additional genetic alterations occur, e.g. loss of tumor suppressor gene CDKN2A [10, 21]. Furthermore, alterations in the PI3K/AKT pathway [22] have been associated with aggressive forms of PA [9]. One proposed mechanism for the often observed slow growth of the tumors is oncogene-induced senescence, which is a mechanism limiting neoplastic growth by inducing cellular senescence. The MAPK activation might initially promote growth as well as induce senescence. Oncogene-induced senescence has also been observed in melanocytic nevi and melanoma [10].

Several mechanisms for tumor regression have been suggested, e.g. immunologic mechanisms, hormonal factors, induction of differentiation or apoptosis [13]. However, the reason why regression in PA occurs is not understood yet [4].

We formulate a mathematical model for growth, progression and regression of PA based on the above described clinical and molecular biological observations. We study the effects of competition between tumor and wild-type cells on the chance for regression. We distinguish two types of PA. Benign cases are classified as *PA-I tumors* and assumed to be caused by alteration of a single pathway. Tumors in which an additional alteration occurs are categorized as *PA- II tumors*, representing the more aggressive subset of PA. We introduce a stochastic tumor growth and progression model, namely a Moran model [23] with mutations. We chose a Moran model in this juvenile tumor, since astrocyte proliferation and diversification mainly happen during late embryogenesis and the first three weeks after birth. These processes are largely complete by early postnatal stages, while early and late postnatal development is mainly characterized by maturation processes (like continuing elaboration of astrocyte processes and building of synaptic/vascular connections) [24, 25]. Since PA are usually diagnosed between 5 and 15 years, the normal astrocyte population is not proliferating at this time anymore. Therefore, it is reasonable to assume an approximately homeostatic tissue. In such a tissue, Moran dynamics provide a natural and established framework for modeling competition between tumor and wild-type cells.

In our model, we derive the PA-regression-function describing the probability for regression in dependency of the residual tumor size after partial resection of benign PA. The accumulation of mutations in a tissue has been modeled and investigated by several authors by using a Moran model. Work by Iwasa, Michor, Komarova and Nowak [26, 27] has been extended by Schweinsberg [28] and durrett, Schmidt and Schweinsberg [29] to the case of *m* mutations. These models analyze tumor growth and progression [30–34] with a focus on theoretical results regarding the waiting time until a cell has accumulated a certain number of mutations. Our approach is motivated by a concrete clinical question which is the regression probability of a benign PA tumor in dependency of the residual tumor size. We modify the model introduced in [29]. In particular, we consider Moran dynamics with two mutations but two absorbing states and investigate the precise relation of the two absorption probabilities which allows the incorporation of epidemiological data to calibrate the model. From the mathematical point of view, the relation of the two absorption probabilities can be connected to the portion of stochastic tunneling events in the model presented in [29].

## Materials and Methods

### Definition of a tumor growth and progression (TGP) model

#### State space and representation of PA tumors

**Fig 1:**
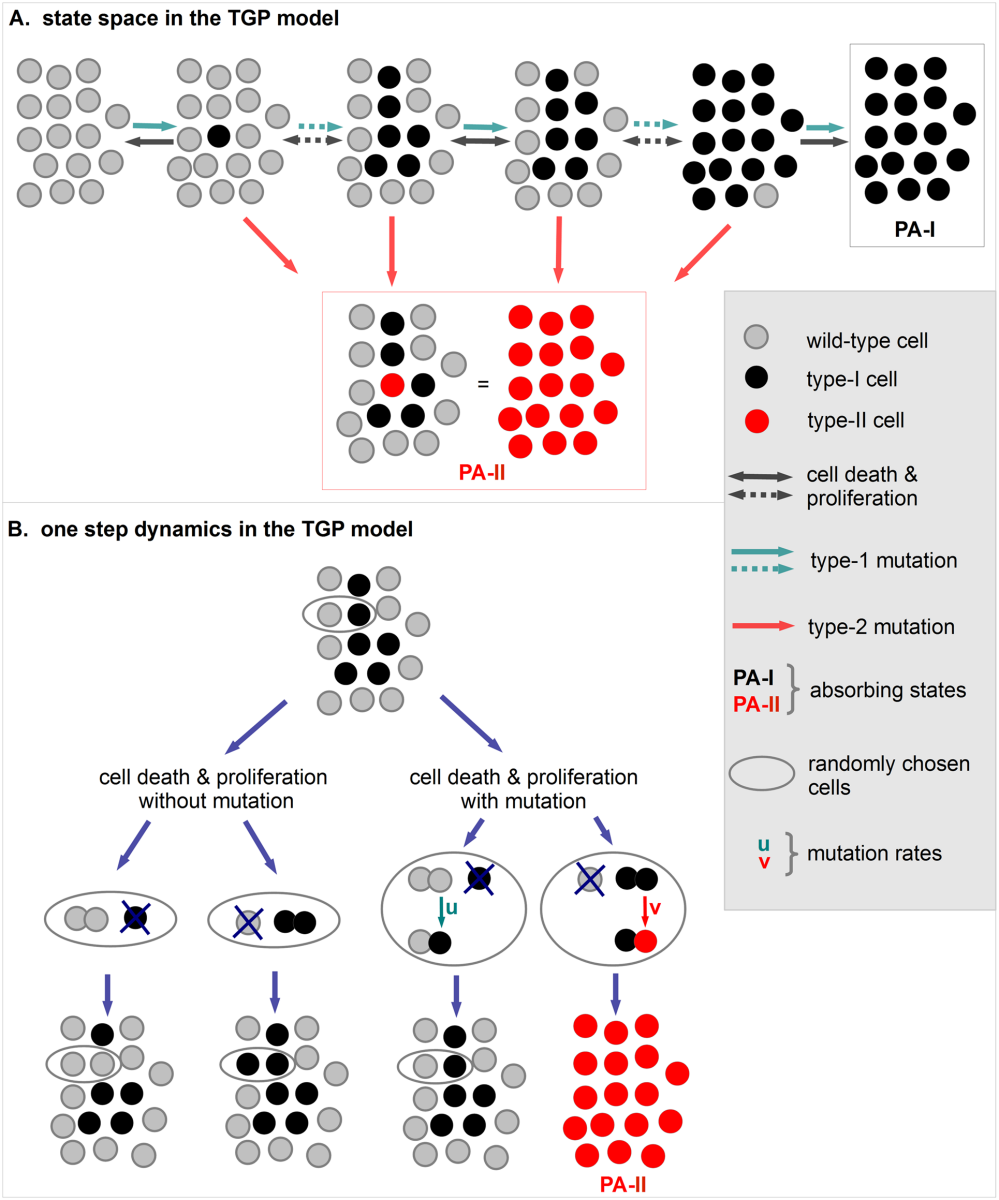
States and dynamics in the TGP model. **A**. The initial state of the TGP model is *all-cells-wild-type*. Type-I cells (black cells) can arise due to type-I mutations. If the number of type-I cells reaches at least the critical tumor size *N*, a PA-I tumor developed which cannot regress anymore (black box). We assume that a PA-II tumor occurs as soon as the first type-II cell appears in the system (red box). Solid arrows indicate a single transition whereas dotted arrows indicate several transitions. **B**. Wild-type cells mutate to type-I cells with probability *u* and type-I cells mutate to type-II cells with probability *v* during proliferation. Cell death and proliferation are included as follows. A cell is randomly chosen for cell death and replaced by the offspring of another randomly chosen cell. Spatial aspects are neglected in the model.

We use a Moran model with mutations to model tumor growth, regression and progression of PA. The model incorporates three cell types, *wild-type cells*, *type-I cells* and *type-II cells*. Wild-type cells have no genetic alteration. Type-I cells are characterized by a MAPK pathway alteration. We assume that type-I cells proliferate without fitness advantage. This is motivated by the observation that benign PA grow very slowly, can be stable in size over a long time period or even regress. Type-II cells have acquired a second genetic alteration disabling oncogene-induced senescence, for example by loss of CDKN2A. Due to their aggressive behavior, we assume a very large fitness advantage of type-II cells. The parameter *N* in our model represents a critical tumor size in the sense that a PA which reaches size *N* cannot spontaneously regress. Our assumption that such a critical size exists is founded on clinical data and observations which are explained later. Here, we focus on the chance of tumor regression of a partially resected benign PA in dependency of the residual tumor size. Therefore, we do not consider growth of tumors beyond the critical size *N* since spontaneous regression would not be possible anymore in such a case. Spatial aspects are neglected so that the number of cells of each cell type is sufficient to describe the states of our model. Therefore, the state space can be described by *S* = {0, 1, 2,...., *N*, *E*}. Here, states 0 to *N* represent the occurrence of the respective number of type-I cells and no type-II cell. The additional state *E* indicates the presence of a type-II cell. States *N* and *E* are absorbing states of the model and represent the occurrence of a benign PA which we call *PA-I tumor* and an aggressive form of PA named *PA-II tumor*, respectively. Hence, the occurrence of a PA-I tumor is represented by the accumulation of *N* type-I cells. As soon as a single type-II cell appears, we identify this state as occurrence of a PA-II tumor which is modeled as absorption in state *E*. Please note that no transition from state *N* to state *E* is possible in the model. Fig 1 illustrates the three cell types and the representation of both types of PA tumors in the model.

#### Dynamics in the model

The dynamics of our model incorporates cell death, proliferation and genetic alterations. Cell death and proliferation are modeled according to the Moran dynamics [23] as follows. Two cells are chosen randomly. One of these cells undergoes cell death and the other cell proliferates. The offspring of the proliferating cell substitutes the cell chosen for death. Since we neglect spatial aspects, the offspring of a cell can replace any other cell. During proliferation, a mutation of the new-born cell can occur. Wild-type cells mutate to type-I cells with probability *u* and these mutate to type-II cells with probability *v*. Moran dynamics is defined with respect to a *relevant cell number* which describes the number of cells that potentially compete with each other. This relevant cell number can be assumed to be approximately equal to the critical tumor size *N* based on the following arguments. Since PA tumors grow as a solid, well-circumscribed mass within the normal brain, new tumor cells are placed in the vicinity of already mutated cells. Similarly, only wild-type cells in the vicinity of mutated cells can potentially compete with tumor cells. Thus, the actual relevant cell number for the Moran dynamics is somewhere between the critical tumor size *N* and the total number of astrocytes in the brain but clearly much closer to *N*. Since there is no detailed experimental estimate of this number so far, we assume in our model that the relevant cell number for Moran dynamics is equal to *N*. Although we do not explicitly incorporate spatial aspects, this assumption implicitly incorporates spatial aspects by implying that tumor cells cannot place its offspring too far away.

We assume that initially all cells are wild-type cells. Hence, the process starts in state 0. The number of type-I cells changes according to the above described dynamics. The precise rates of the process are provided in S1 Text and the dynamics are illustrated in Fig 1.

The described model is called *TGP process* and the corresponding stochastic process is denoted by (*X*_*t*_)_*t* ≥ 0_. The TGP process is a Markov process with two absorbing states *N* and *E* representing a PA-I tumor and a PA-II tumor, respectively. Hence, the absorption probabilities of the TGP process in both states correspond to the clinically observed fraction of PA-I and PA-II tumors. Therefore, we will derive these absorption probabilities and analyze in which way they depend on the model parameters *N*, *u* and *v*. Furthermore, we assume that tumor regression is characterized by the vanishing of all tumor cells. Hence, tumor regression corresponds to reaching state 0 in the TGP model which will be described by a tumor regression function in the following.

### Analysis of the TGP process

The behavior of the TGP process depends on its three parameters, the critical tumor size *N*, the mutation probability from wild-type cells to type-I cells *u* and the mutation probability from type-I cells to type-II cells *v*. The parameter regime for the analysis of the TGP model is chosen such that

$$\begin{array}{c} u\ll \frac{1}{N} \end{array}$$ (1)

and

$$\begin{array}{c} {\left(N\sqrt{v}\right)}^{2}=:\gamma >0. \end{array}$$ (2)

In the following we explain this choice. We call the parameter *γ* *risk coefficient*.

#### Decomposition of the TGP process into two sub-processes

**Fig 2:**
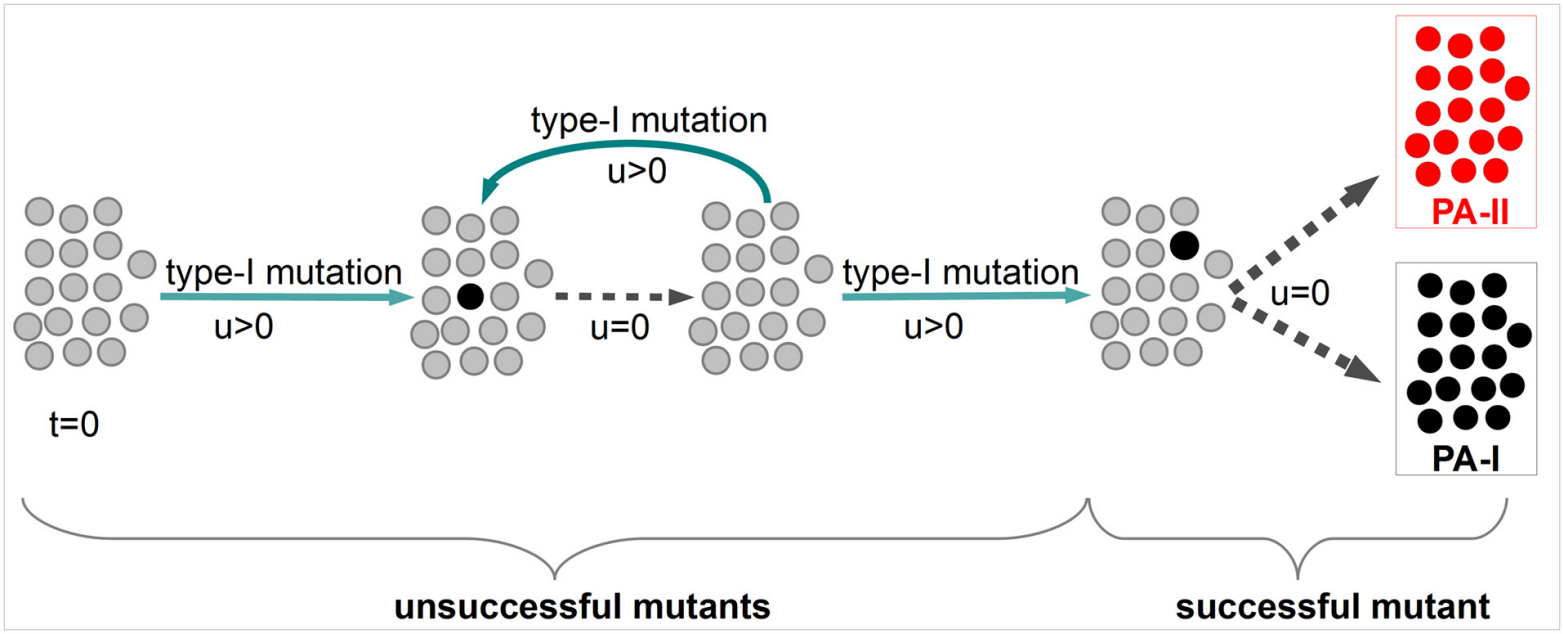
Decomposition of the TGP process. Assumption Eq (1) implies that no other PA-I mutation occurs if type-I cells are already present in the system. Therefore, *u* can be set to zero when a single type-I cell emerged. This allows to decompose the process into two sub-processes. First, occurrence of unsuccessful mutants, which go extinct and, second, the occurrence of a successful mutant which leads to absorption in one of the PA states.

Assumption Eq (1) implies that type-I mutations are rare. Typically, an emerging type-I lineage either goes extinct or leads to absorption of the TGP process before another type-I mutation occurs. Hence, each newly arising type-I mutant can be investigated independently. This idea has been introduced in [29]. Therefore, we set *u* = 0 as soon as type-I cells are present, i.e. if the TGP process is in state *k*, 1 ≤ *k* ≤ *N*. Since absorption is inevitable in the TGP model, a *successful* type-I mutant that leads to absorption in state *N* or state *E* must eventually occur. Before the occurrence of this specific type-I mutant, *unsuccessful* mutants arise and go extinct driving the process back to state 0. Hence, assumption Eq (1) implies that a PA tumor develops from a single mutated cell. See also Fig 2 for an illustration of this decomposition of the TGP model.

#### Absorption probability of the TGP process

As Fig 2 illustrates, the absorption probabilities of the TGP model agree with those of the sub-process starting with emergence of the successful mutant. This sub-process is formally defined in S1 Text, where also a detailed derivation of the absorption probability in state *N* of the TGP model is provided. This derivation utilizes first step analysis in order to obtain a linear system of equations for the absorption probabilities starting with *k*, 1 ≤ *k* ≤ *N*, type-I cells. Subsequently, Cramer’s rule, see equation (S4) in (S1 Text), allows to derive the particular absorption probability starting with one type-I mutant. Taking the limit for *N* → ∞ leads to the *asymptotic absorption probability in state N* given by

$$\begin{array}{c} \alpha \left(\gamma \right)=\frac{1}{I_{0}\left(2\sqrt{\gamma }\right)}, \end{array}$$ (3)

where $I_{n},n\in {\text{I}\;\text{N}}_{0},$ denote the modified Bessel functions of the first kind, see [35]. A plot of *α*(*γ*) is given in S1 Fig. The absorption probability *α*(*γ*) corresponds to the fraction of PA-I tumors in the TGP model. Three different behaviors regarding absorption can be distinguished in dependency of the parameters *N* and *v*.

1. *If* $\sqrt{v}\gg \frac{1}{N}$, *then the system is primarily absorbed in state E*. The assumption guarantees that the number of type-I cells is only a small fraction of the critical number of mutated cells *N* throughout the process so that the probability of reaching state *N* can be neglected. The probability that a single arising type-I cell reaches fixation is approximately $\frac{1}{N}$. This is much smaller than $\sqrt{v}$, which is asymptotically the probability that this cell produces a type-II cell before going extinct.
2. *If* $\sqrt{v}⪡\frac{1}{N}$, *then the system is primarily absorbed in state N*. In this case, it is unlikely that a type-II cell appears before the system reaches state *N* so that the probability of reaching state *E* vanishes.
3. *If* ${\left(N\sqrt{v}\right)}^{2}=\gamma >0$, *then the system can be absorbed both in state N and in state E*. In this case a mutation to a type-II cell occurs with positive probability before state *N* is reached.

For rigorous proofs of these results see [29] and for a good sketch of the proofs without technical details see [36].

To model growth and progression of PA, we focus on the third case since this parameter regime implies strictly positive absorption probabilities in both absorbing states *N* and *E*. Hence, both PA-I and PA-II tumors occur with positive probability in the model which justifies assumption Eq (2).

### Derivation of tumor regression functions in the TGP model

**Fig 3:**
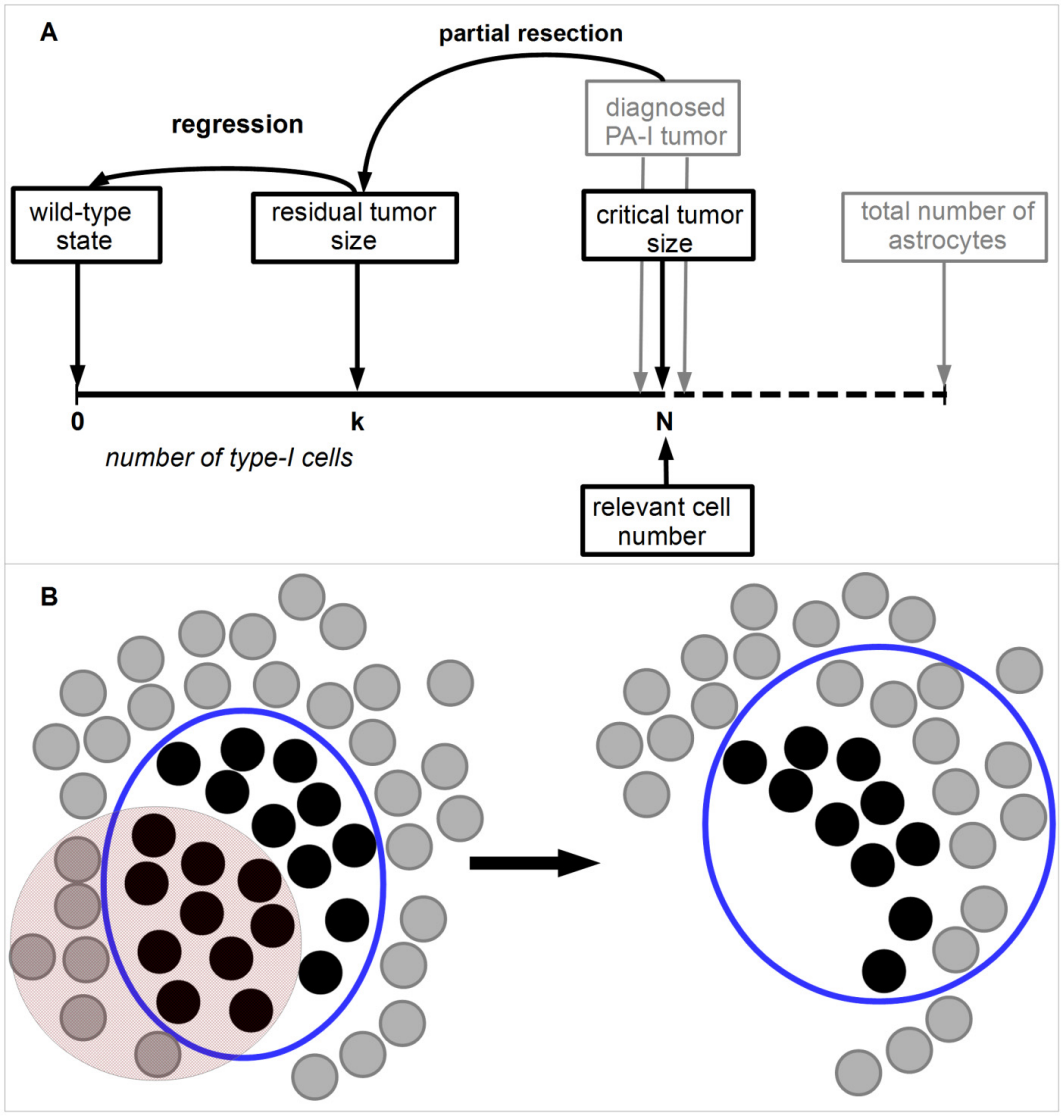
Tumor regression in the TGP model. **A**. Partial resection of a PA-I tumor reduces the number of tumor cells to size *k* which is assumed to be below the critical tumor size *N*. The residual tumor can regrow, progress or regress based on the same dynamics that led to the primary tumor. Hence, the TGP dynamics with relevant cell number *N* is utilized to describe the further development of the residual tumor. Regression is achieved if state 0 is reached, i.e. no tumor cells are present anymore. **B**. The red area indicates the resected part of the diagnosed PA-I tumor. This resection leads to removal of both tumor and wild-type cells. Subsequently, the residual number of tumor cells *k* competes with other wild-type cells which can lead to regrowth, regression or progression of the residual tumor. We assume that *N* is the relevant cell number for this competition as in the formation of the primary tumor. This relevant cell number is indicated by the blue circle.

We are interested in the regression probability of a partially resected PA-I tumor in dependency of the remaining tumor size and assume that regression of a residual tumor is achieved if no tumor cells are present anymore. All suggested mechanisms of tumor regression influence the ratio of tumor and wild-type cell birth and death rates. Therefore, we assume that competition between tumor and wild-type cells leads to tumor regression which is incorporated by Moran dynamics with relevant cell number equal to *N* again, see also Fig 3. Furthermore, we assume that the partial resection reduces the residual number of PA-I cells below the critical tumor size *N*. Hence, the regression function is defined as the extinction probability of tumor cells, i.e. the probability to reach state 0 when starting the TGP process in some state *k* with 1 ≤ *k* ≤ *N* − 1. For *v* = 0, our TGP process simplifies to a neutral two-type Moran process in which the extinction probability is an established result and equals $1-\frac{k}{N}$ [32]. Here, we derive this extinction probability for our TGP process with three cell types. For the mathematical analysis, it is convenient to express this function in terms of $\rho =\frac{k}{N}$. The fraction *ρ* describes the ratio between the residual number of PA-I cells after partial resection *k*, 1 ≤ *k* ≤ *N* − 1, and the critical tumor size *N*.

Formally, these considerations lead to the regression function $\beta_{\gamma }^{N}\left(\rho \right)$ defined as

$$\begin{array}{c} \beta_{\gamma }^{N}\left(\rho \right):=\text{I}\;\text{P}\left(X_{t}=0\;\text{for}\;\text{some}\;t\geq 0|X_{0}=N\rho \right),\;\rho \in \left[0,1\right]. \end{array}$$ (4)

Fig 3 provides a graphical representation of regression in the TGP model.

**Fig 4:**
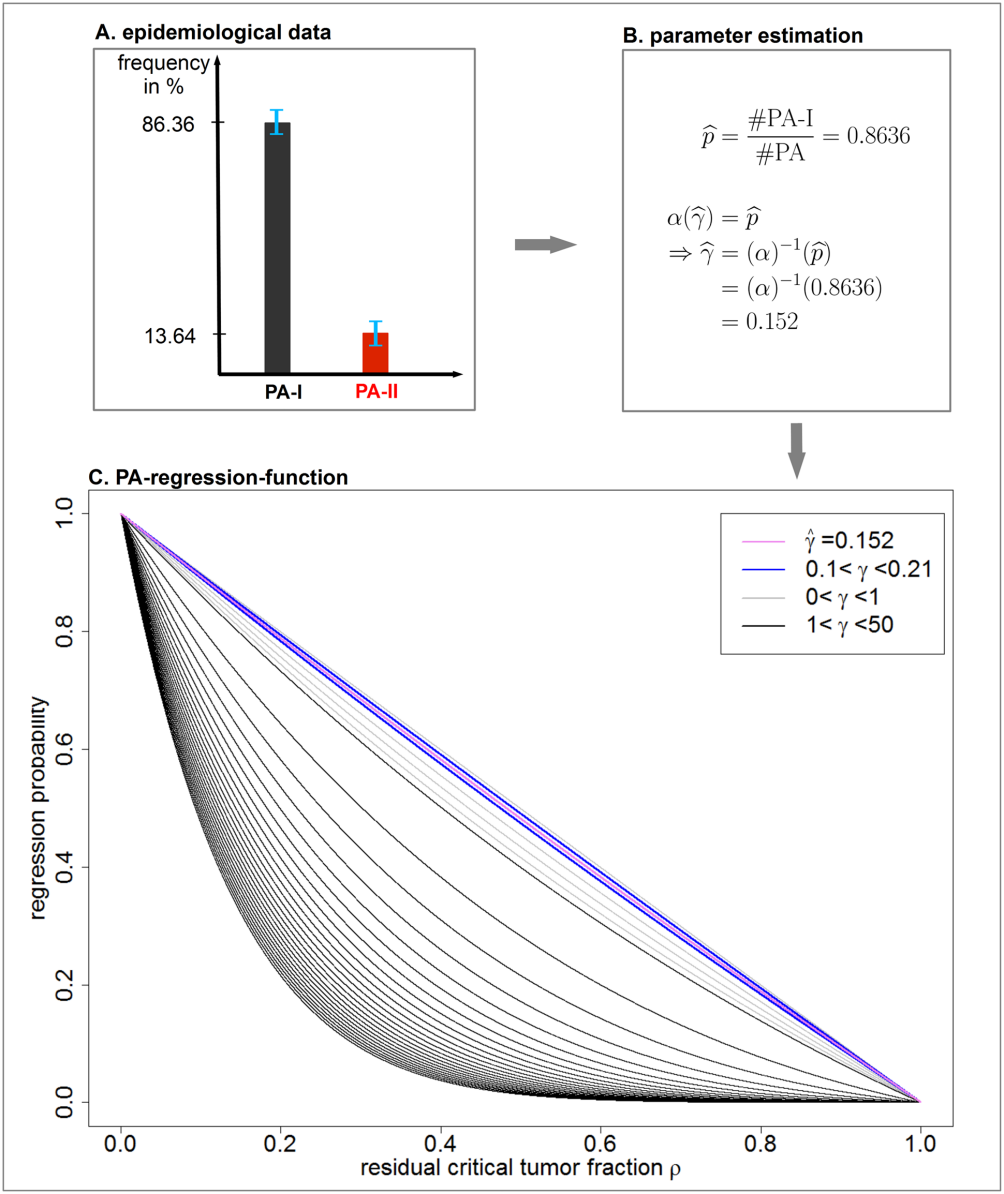
Parameter estimation from epidemiological data and derivation of the PA-regression-function. **A**. In order to estimate the clinically observed fraction of PA-I, we utilize data from the literature. **B**. The estimated fraction of PA-I cases $\hat{p}$ is interpreted as absorption probability in state *N* in our model. Eq (3) allows to estimate the corresponding risk coefficient $\hat{\gamma }$. **C**. Substituting the estimated risk coefficient $\hat{\gamma }=0.152$ in Eq (5) determines the PA-regression-function *β*_0.152_(*ρ*) which is plotted in purple. Furthermore, the blue plots indicate the corresponding regression functions for values of *γ* which are obtained within the standard deviation of $\hat{p}$.

A diffusion approximation of (*X*_*t*_)_*t* ≥ 0_ leads to the Wright-Fisher diffusion process that can be utilized to approximate the term of Eq (4). This approach was introduced in [29] and leads finally to a series representation as approximation of $\beta_{\gamma }^{N}\left(\rho \right)$. In S1 Fig it is shown that this series can be expressed by Bessel functions $I_{n},n\in \text{I}\;\text{N},$ [35] and that the regression function of the TGP model is given by

$$\begin{array}{c} \beta_{\gamma }\left(\rho \right)=\frac{\sqrt{1-\rho }I_{1}(2\sqrt{\gamma (1-\rho )})}{I_{1}\left(2\sqrt{\gamma }\right)} \end{array}$$ (5)

for 0 ≤ *ρ* ≤ 1. The graph of *β*_*γ*_ is plotted in Fig 4C for different values of the risk coefficient *γ*.

#### Taylor expansion of the tumor regression function

In order to estimate the deviation of the specific tumor regression function for PA from a linear function, we derive the first order Taylor polynomial *T*_1_(*ρ*) at *ρ* = 0.5 of the regression function Eq (5) and an estimation of the remainder term *R*_1_(*ρ*). It holds that

$$\begin{array}{ccc} T_{1}\left(\rho \right) & = & \frac{I_{1}(\sqrt{2\gamma })}{\sqrt{2}I_{1}(2\sqrt{\gamma })}-\frac{\sqrt{2}I_{1}(\sqrt{2\gamma })+\sqrt{\gamma }(I_{0}(\sqrt{2\gamma })+I_{2}(\sqrt{2\gamma }))}{2I_{1}(2\sqrt{\gamma })}\left(\rho -0.5\right),\\ |R_{1}\left(\rho \right)| & \leq & \frac{\gamma }{8}, \end{array}$$ (6)

for *ρ* ∈ [0, 1]. The Supplementary Information, (S1 Text), provides a detailed derivation.

## Results

### Derivation of the PA-regression-function

The regression function Eq (5) depends on the parameters of the TGP model via the risk coefficient *γ*, see Eq (2). This parameter is estimated such that the clinically observed fraction of PA-I tumors, denoted by $\hat{p}$, equals the theoretically obtained fraction *α*(*γ*) of absorption in state *N* in the TGP model. Subsequently, the derived risk coefficient is substituted into the regression function given by Eq (5) in order to obtain the specific PA-regression-function. Fig 4 summarizes the overall strategy of this approach.

We estimate the clinically observed fraction of PA-I tumors on the basis of data reported in [10]. The authors analyzed 66 PAs with respect to their genetic profile and classified 57 cases as benign PA-I tumors and 9 cases as more aggressive PA-II tumors. This leads to

$$\hat{p}=\frac{57}{66}=0.8636.$$

In the TGP model, this clinically observed fraction corresponds to the absorption probability in state *N*, given by Eq (3). Therefore, we set

$$\alpha (\hat{\gamma })=\hat{p}=0.8636.$$

This equation allows to calculate the risk coefficient $\hat{\gamma }$ which yields

$$\begin{array}{c} \hat{\gamma }=0.152. \end{array}$$

Substituting $\hat{\gamma }=0.152$ into the regression function given by Eq (5) allows to derive the *PA-regression-function* given by

$$\begin{array}{c} \beta_{0.152}\left(\rho \right)=2.3795\sqrt{1-\rho }I_{1}(0.7797\sqrt{1-\rho }),\;0\leq \rho \leq 1. \end{array}$$ (7)

A plot of this function is provided in Fig 4C. This figure shows that the regression function is very robust to small alterations with respect to $\hat{p}$.

Note that the actual risk coefficient may be smaller than the estimated value $\hat{\gamma }=0.152$ due to the following considerations. The parameter *N* in our model represents a critical tumor size above which tumor regression cannot be expected anymore. However, the number of mutated cells in a diagnosed PA-I tumor may be larger than *N* because tumors could grow beyond this critical size without symptoms or due to a diagnostic gap between first symptoms and diagnosis. Therefore, a PA-I tumor can consist of more than *N* type-I cells and should have been more susceptible for progression to PA-II than accounted for in our TGP model. Hence, the risk of progression in our TGP model and therefore $\hat{\gamma }$ might be overestimated. However, this would not change the linear dependency between residual tumor size and regression probability which is discussed in the following.

### Linear dependency between residual tumor fraction and regression probability of PA

We can show that the PA-regression-function Eq (7) is approximately linear by utilizing a Taylor expansion using Eq (6). Substituting the estimated risk coefficient of the PA-regression-function $\hat{\gamma }=0.152$ into Eq (6) leads to

$$\begin{array}{c} T_{1}\left(\rho \right)=0.9817-\rho ,\;\rho \in \left[0,1\right]. \end{array}$$ (8)

This is a very good approximation since the remainder term can be estimated by

$$\begin{array}{c} |R_{1}\left(\rho \right)|\leq \frac{\gamma }{8}=\frac{0.152}{8}=0.0185 \end{array}$$ (9)

for *ρ* ∈ [0, 1]. Hence, the deviation of the PA-regression-function from the linear function *T*_1_(*ρ*) is very small. Moreover, if the risk coefficient was overestimated, an even smaller deviation would be observed as Eq (9) implies.

### Quantitative prediction of the regression probability for benign PA

In order to provide a quantitative prediction of the regression probability given the absolute residual tumor size, we estimate the critical tumor size *N* in our model. Since the total cell number corresponds to the the tumor volume, we can interpret *N* also as minimum absolute tumor volume above which tumor regression cannot be expected anymore. The existence of this critical tumor size and its estimate of a cell number corresponding to a volume of 9 cm^3^ is justified in the following way. First, an extensive literature research indicated that tumor regression for residual cerebellar PA over 9 cm^3^ has not been reported yet, see S1 Table. Second, the prediction for patients with 78 cerebellar astrocytoma, including 62 PAs, has been investigated in [15]. Fig. 6 in [15] implies that the theoretical proportion of progression-free patients based on a Cox regression analysis with a residual tumor of 9 cm^3^ is estimated to be zero in the long-term. Finally, in [18], the role of the extent of resection in the long-term outcome of low-grade gliomas is investigated including 93 PAs. It is stated that “‘*the predicted outcome for patients is negatively influenced by even residual tumor volumes on the order of 10 cm^3^*”’.

**Fig 5:**
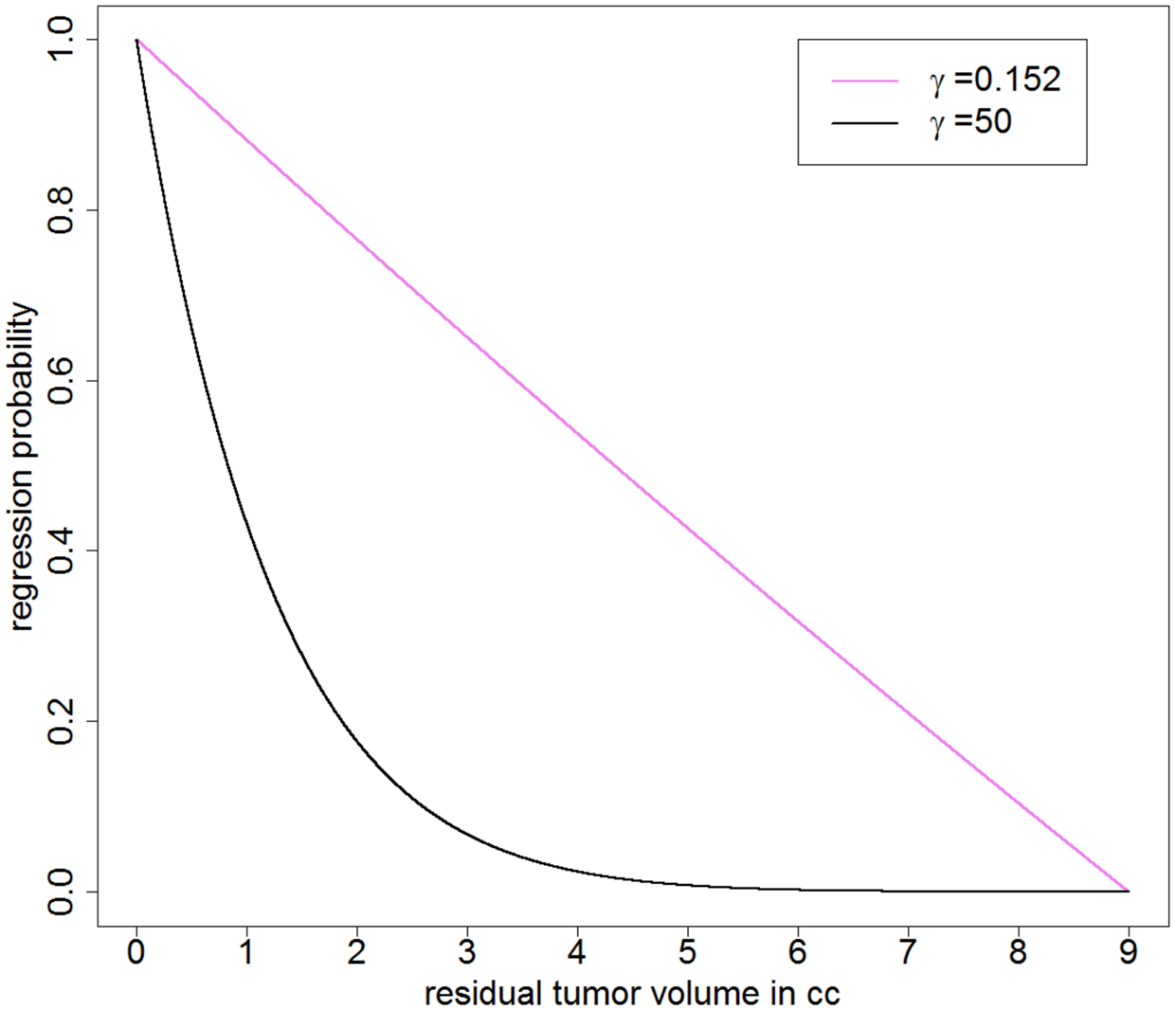
Non-existence of an EOR threshold in PA. The derived PA-regression-function (purple line) allows to quantitatively predict the regression probability based on the critical tumor size estimated as 9 cm^3^, see also Table 1. Roughly, one cm^3^ of resected tumor mass will elevate the chance of regression by 10%. The direct consequence is the non-existence of an EOR threshold implying that any proportion of resected tumor mass will improve prognosis. This stands in contrast to the behavior of the regression function for a fictive high value of the risk coefficient of e.g. *γ* = 50 (black line).

Incorporating the estimation for the critical tumor size of 9 cm^3^ into the PA-regression-function Eq (7) allows to quantify our predictions, indicating that any volume reduction of one cm^3^ below the critical size will add 10% to the chance for regression (see also Fig 5 and Table 1).

**Table 1:** Predicted regression probability for cerebellar PA based on the absolute residual tumor size.

Residual tumor size (cm^3^)	Tumor regression probability (in %)
0.1	98.91
0.5	94.06
1	88.16
2	76.50
3	65.03
4	53.75
5	42.64
6	31.71
7	20.47
8	10.39

The values in this table are derived from the PA-regression-function provided by Eq (7) and use an estimate for the critical tumor size of 9 cm^3^. The PA-regression-function is also illustrated in Fig 5.

### Non-existence of an extent of resection (EOR) threshold

In malignant brain tumors it has been shown that there is an EOR threshold below which no survival advantage is provided, e.g. in glioblastoma this threshold is 78% [37]. The existence of different tumor zones which basically reflect tumor heterogeneity is one proposed reason for such a threshold in malignant brain tumors [38]. In contrast, our results suggest the non-existence of such a threshold in PA. This is an immediate consequence of the linear dependency between residual tumor size and regression probability. If the residual tumor is smaller than the critical tumor size *N*, which marks the volume for which regression cannot be expected anymore, any reduction of the tumor volume will contribute to the regression probability. Importantly, this behavior stands in contrast to a non-linear dependency which would have been obtained in our model for a higher estimated risk coefficient *γ*, see Fig 5.

## Discussion

In order to gain insights into the regression behavior after partial resection of benign PA, we introduced a stochastic TGP model based on recent molecular findings, functional, and clinical data. We derived a regression function that depends on the risk coefficient *γ* and quantifies the probability of regression in dependency of the residual tumor size. By incorporating epidemiological data on the clinically observed fractions of PA-I and PA-II cases, we estimated *γ* and derived the specific PA-regression-function, given by Eq (7). The estimated PA-regression-function implies an approximately linear dependency between the residual critical tumor fraction and the regression probability as illustrated in Fig 4C. This linear dependency is supported by a Taylor approximation and an estimation of the remainder term, given by Eqs (8) and (9), respectively. Furthermore, we quantitatively predicted the chance for tumor regression for benign PA by estimating the critical tumor size *N*, see Table 1.

Our TGP model incorporates assumptions based on clinical observations. It is observed in the clinics that PA-I tumors grow slowly, arrest in growth, or even regress. Hence, type-I cells in our model proliferate without fitness advantage. Furthermore, we assume that the first type-II cell that occurs leads to an aggressive form of PA, corresponding to malignant progression in PA. Alternatively, one could assign a success probability *s* to an emerging type-II cell, which represents the probability that a single type-II cell leads to a PA-II tumor. However, it has been shown in [36] that this is equivalent to considering an analog process with type-II mutation probability *sv* instead of *v*. This alternative process would lead to the same estimated risk coefficient $\hat{\gamma }$. Therefore, the estimated PA-regression-function would not change since this function is determined only by $\hat{\gamma }$. Further, we use asymptotic results for *N* → ∞ in order to calculate the theoretical portion of PA-I and PA-II cases in the TGP model. This is justified by the fact that a tumor consists of billions of cells. Simulation results given in S2 Table support these asymptotic results. They show that excellent accordance with formulas for finite *N* is reached even for small values of *N*. Moreover, we could show that the model is robust against small changes in the proportion of PA-I versus PA-II tumors as shown in Fig 4C. This robustness is an important property of the model since the proportion of PA-I can vary between different studies, especially since the sample size is often very small [39, 40].

To our knowledge, the proposed TGP model is the first theoretical attempt to predict the regression behavior of PA. In particular, we analyzed PA regression based on the population dynamics of tumor and wild-type cells. The ratio of tumor cell birth and death rates is influenced by immunologic mechanisms, hormonal factors, induction of differentiation, or apoptosis, which could all contribute to tumor regression [13]. Since PA-I tumors grow very slowly, we assumed identical birth and death rates of type-I cells in our model.

Our findings have clinically relevant implications. There is still controversy about the best treatment strategy for PA. Since PA is a slowly growing tumor and might even spontaneously regress, a wait and see strategy is an option besides more aggressive treatment strategies like radiation and chemotherapy. The decision for a more radical therapy would depend on the risk for recurrence (or even progression) and the chance of regression. However, long-term follow-up data about the probability of regression or progression after partial resection of PA is restricted and only retrospective studies with small case numbers are available [39–42]. The linear dependency between residual tumor size and regression probability in our model implies that every resected percentage point of a PA-I tumor contributes equally to the regression probability. Hence, there is no EOR threshold, but any small reduction in tumor mass provides an improvement in prognosis by increasing the probability for tumor regression. This prediction suggests a fundamentally different treatment strategy for PA compared to glioblastoma for which such a threshold has been determined [37]. Therefore, our results indicate that resection of a tumor should be aimed at even if a complete resection may not be possible. This is supported by studies showing that in patients with PA outcome depends on the extent of resection, although these studies only differentiate between *biopsy, partial, subtotal*, and *gross/total* resection and do not measure tumor volumes [3, 15, 16]. Moreover, if complete resection cannot be achieved, our results predict that the outcome linearly depends on the residual tumor volume. If there is a reasonable chance for regression of the residual tumor, it might be less justified to accept side effects by further therapies like radiation. This is an important result since the role of additional radiation therapy in treating children with tumors is highly controversial [8]. Unfortunately, as far as we know, there are no clinical studies on treatment of PA that take into account the influence of the residual tumor volume on patient outcome. We suggest that the residual tumor volume is an important prognostic marker and that a lack of sufficient volumetric data could be a reason for different results in clinical studies on additional treatment in PA.

The results of this work should be further supported by future clinical studies that include volumetric data, which will improve the quantitative prediction of our model and form a statistical basis for clinical decision rules.

## Supporting Information

S1 Text Supplementary information. The supplementary text contains detailed information about: 1) transition rates of the TGP model; 2) detailed derivation of the absorption probabilities; 3) derivation of the asymptotic absorption probabilities of the TGP model; 4) derivation of the regression function; 5) Taylor expansion of the regression function.
(PDF)

S1 FigThe asymptotic absorption probability in state *N* in dependency of the risk coefficient *γ*.The asymptotic absorption probability *α*(*γ*) in state *N* is strictly monotonically decreasing. The clinically observed fraction of PA-I cases is estimated as $\hat{p}=0.8634$ and the corresponding risk coefficient is $\hat{\gamma }=0.152$.(TIF)

S1 Table Residual cerebellar postoperative PA volume and outcome. This table contains the results of our literature research about volumetric data of residual cerebellar PA and the corresponding patient outcome.
(PDF)

S2 Table Comparison of theoretical absorption probabilities and simulation results. This table shows the exact absorption probabilities *α* ^*N*^(*γ*) in state *N* compared to the results from 10000 simulations of trajectories of the TGP process (*X*_*t*_)_*t* ≥ 0_ and the asymptotic absorption probabilities *α*(*γ*). The asymptotic values are in good accordance with both, the exact theoretical values and the simulation results. Furthermore, the asymptotic result *α*(*γ*) is a good approximation even for small *N*.
(PDF)
