# Supplementary material for: Model-Based Evaluation of Spontaneous Tumor Regression in Pilocytic Astrocytoma
Source: PLoS Comput Biol. 2015 Dec 10;11(12):e1004662. doi: 10.1371/journal.pcbi.1004662 (PMC4675550; doi:10.1371/journal.pcbi.1004662)
Supplement: S1 Text — The supplementary text contains detailed information about: 1) transition rates of the TGP model; 2) detailed derivation of the absorption probabilities; 3) derivation of the asymptotic absorption probabilities of the TGP model; 4) derivation of the regression function; 5) Taylor expansion of the regression function. (PDF) [file pcbi.1004662.s001.pdf]

Supplementary Material

Transition rates

Formally, the TGP process $(X_t)_{t\geq 0}$ is modeled as a MARKOV process on the state space $S = \{0, 1, 2, ..., N, E\}$. The dynamics is determined by the rate matrix $Q = (q(k,l))_{k,l\in S}$ with

$$q(k,l) = \begin{cases} \frac{(N-k)k}{N}(1-v) + \frac{(N-k)(N-k)u}{N}, & 0 \leq k \leq N-1, l = k+1, \\ \frac{(N-k)k(1-u)}{N}, & 1 \leq k \leq N-1, l = k-1, \\ kv, & 1 \leq k \leq N-1, l = E, \\ -\sum\limits_{\substack{m\in S \\ m\neq k}} q(k,m), & l = k, \\ 0, & \text{else}, \end{cases} \tag{S1}$$

where u and v represent the mutation probabilities from wild-type to type-I cells and from type-I cells to type-II cells, respectively. These rates induce that the states N and E are absorbing states of the process.

Absorption probabilities

In order to calculate the absorption probabilities of the MARKOV process determined by the rates in equation (S1), a sub-process is investigated. This sub-process is characterized by the state space $\widetilde{S} = \{1, 2, 3, ..., N, E\}$ and rate matrix $\widetilde{Q} = (\widetilde{q}(k,l))_{k,l\in S}$ which is obtained from the original rates given in (S1) by eliminating state 0 and setting $u = 0$ such that

$$\widetilde{q}(k,l) = \begin{cases} \frac{(N-k)k}{N}(1-v), & 1 \leq k \leq N-1, l = k+1, \\ \frac{(N-k)k}{N}, & 2 \leq k \leq N-1, l = k-1, \\ kv, & 1 \leq k \leq N-1, l = E, \\ -\sum\limits_{\substack{m\in \widetilde{S} \\ m\neq k}} q(k,m), & l = k, \\ 0, & \text{else}. \end{cases} \tag{S2}$$

By defining $\widetilde{q}(k) := -\widetilde{q}(k,k)$, we get

$$\widetilde{q}(1) = \widetilde{q}(1,E) + \widetilde{q}(1,2) = \frac{N+v-1}{N},$$

$$\widetilde{q}(k) = \widetilde{q}(k,k+1) + \widetilde{q}(k,k-1) + \widetilde{q}(k,E) = \frac{2(N-k)k + k^2 v}{N}, \quad 2 \leq k \leq N-1.$$

We further define transition probabilities

$$p(i,j) := \begin{cases} \frac{\widetilde{q}(i,j)}{\widetilde{q}(i)}, & i \neq j, \\ 0, & \text{else}, \end{cases}$$

which look as follows.

$$\begin{aligned}
p(1,E) &= \frac{Nv}{N+v-1}, \\
p(1,2) &= \frac{(N-1)(1-v)}{N+v-1}, \\
p(k,E) &= \frac{Nv}{2(N-k)+kv}, \quad 2 \leq k \leq N-1, \\
p(k,k+1) &= \frac{(N-k)(1-v)}{2(N-k)+kv}, \quad 2 \leq k \leq N-1, \\
p(k,k-1) &= \frac{(N-k)}{2(N-k)+kv}, \quad 2 \leq k \leq N-1, \\
p(E,E) &= 1.
\end{aligned}$$

The absorption probabilities of the processes determined by the rate matrix q and the transition matrix P are equal. This holds due to the fact that P implies an equivalent process where we only eliminated transitions into the same state which only influences the time-scale of the process. The absorption probabilities for the process which is determined by the transition matrix $P = (p_{i,j})_{i,j \in \widetilde{S}}$ is obtained as follows. Denote by the vector $\alpha^N = \big(\alpha^N(i,v)\big)_{i \in \widetilde{S}}$ the absorption probabilities, where $\alpha^N(i,v)$ equals the absorption probability in state N starting from state i. First step analysis yields

$$\alpha^N(i,v) = \sum_{j \in \widetilde{S}} p(i,j)\alpha^N(j,v), \quad i \in \widetilde{S}.$$

It holds that $\alpha^N(E,v)=0$, $\alpha^N(N,v)=1$ and therefore

$$\begin{aligned}
\alpha^N(i,v) &= \sum_{j=1}^{N} p(i,j)\alpha^N(j,v) \\
&= \sum_{j=1}^{N-1} p(i,j)\alpha^N(j,v) + p(i,N) \\
&= p(i,i-1)\alpha^N(i-1,v) + p(i,i+1)\alpha^N(i+1,v) + p(i,N) \\
&= \begin{cases} \frac{(N-1)(1-v)}{N+v-1}\alpha^N(2,v), & i=1 \\ \frac{(N-i)}{2(N-i)+iv}\alpha^N(i-1,v) + \frac{(N-i)(1-v)}{2(N-i)+iv}\alpha^N(i+1,v), & 2\le i\le N-2, \\ \frac{1}{2+(N-1)v}\alpha^N(N-2,v) + \frac{1-v}{2+(N-1)v}, & i=N-1. \end{cases}
\end{aligned}$$

Hence,

$$\begin{aligned}
-\alpha^N(1,v) + \frac{(N-1)(1-v)}{N+v-1}\alpha^N(2,v) &= 0 \\
\frac{(N-i)}{2(N-i)+iv}\alpha^N(i-1,v) - \alpha^N(i,v) + \frac{(N-i)(1-v)}{2(N-i)+iv}\alpha^N(i+1,v) &= 0, \quad 2\le i\le N-2, \\
-\alpha^N(N-1,v) + \frac{1}{2+(N-1)v}\alpha^N(N-2,v) &= -\frac{1-v}{2+(N-1)v}.
\end{aligned}$$

By multiplying each line with its denominator, one gets an equivalent system $P'\widetilde{\alpha}^N = b$ for a $(N-1)\times(N-1)$ matrix P' and $\widetilde{\alpha}^N := (\alpha^N(i,v))_{i=1,\ldots,N-1}$. This system reads in tableau form as follows.

	$\alpha^N(1,v)$	$\alpha^N(2,v)$	$\alpha^N(3,v)$	...	$\alpha^N(N-1,v)$	1
1	$-(N+v-1)$	$(N-1)(1-v)$	0	$\cdots$	0	0
2	$(N-2)$	$-2(N-2)-2v$	$(N-2)(1-v)$	$\ddots$	0	0
3	0	$(N-3)$	$-2(N-3)-3v$	$\ddots$	$\vdots$	$\vdots$
$\vdots$	$\vdots$	$\ddots$	$\ddots$	$\ddots$	0	$\vdots$
$\vdots$	$\vdots$	$\ddots$	$\ddots$	$\ddots$	$2(1-v)$	0
$N-1$	0	$\cdots$	$\cdots$	1	$-2-(N-1)v$	$-(1-v)$

(S3)

We are interested in the absorption probability $\alpha^N(1,v)=\widetilde{\alpha}^N(1,v)$, i.e. the probability of getting absorbed in state N when the process is started with a single type-I cell. We

use CRAMER'S rule which reads

$$\alpha^N(1,v) = \frac{\det P_1^{'}}{\det P'}, \tag{S4}$$

where $P_1^{'}$ is the matrix formed by replacing the first column of $P^{'}$ by the column vector b. We calculate det $P^{'}$ first. By induction over N the general structure can be inferred. It holds that

$$\begin{aligned}\det P^{'} = \begin{vmatrix} -(3+v) & 3(1-v) & 0 \\ 2 & -4-2v & 2(1-v) \\ 0 & 1 & -2-3v \end{vmatrix} &= 6(v^3+9v^2+9v+1) \\ &= 3!(v^3+3^2v^2+3^2v+1), \text{ for } N=4 \text{ and}\end{aligned}$$

$$\begin{aligned}\det P^{'} = \begin{vmatrix} -(4+v) & 4(1-v) & 0 & 0 \\ 3 & -6-2v & 3(1-v) & 0 \\ 0 & 2 & -4-3v & 2(1-v) \\ 0 & 0 & 1 & -2-4v \end{vmatrix} &= 24(v^4+16v^3+36v^2+16v+1) \\ &= 4!(v^4+4^2v^3+6^2v^2+4^2v+1) \text{ for } N=5.\end{aligned}$$

Furthermore,

$$\begin{aligned}\det P^{'} &= 120(v^5+25v^4+100v^3+100v^2+25v+1) \\ &= 5!(v^5+5^2v^4+10^2v^3+10^2v^2+5^2v+1) \text{ for } N=6.\end{aligned}$$

Therefore, we conclude that the general form of det $P^{'}$ is given by

$$\begin{aligned}\det P^{'} &= (N-1)!\left(\binom{N-1}{N-1}^2 v^{N-1} + \binom{N-1}{N-2}^2 v^{N-2} + \ldots + \binom{N-1}{1}^2 v^1 + \binom{N-1}{0}^2 v^0\right) \\ &= (N-1)!\sum_{i=0}^{N-1}\binom{N-1}{i}^2 v^i \\ &= (N-1)!P_{N-1}\left(\frac{v+1}{1-v}\right)(1-v)^{N-1}, \quad N \in \mathbb{N}. \end{aligned} \tag{S5}$$

Here, $P_N(x)$ denotes the LEGENDRE polynomials [2] which are the particular solutions to the LEGENDRE differential equation

$$\left(1-x^2\right) f''(x) - 2x\, f'(x) + N(N+1)\, f(x) = 0, \quad N \in \mathbb{N}_0.$$

The second determinant is calculated as follows. The matrix P_1' has the structure

$$P_1' = \begin{pmatrix} 0 & (N-1)(1-v) & 0 & \cdots & \cdots & 0 \\ 0 & -2(N-2)-2v & (N-2)(1-v) & 0 & \ddots & 0 \\ 0 & (N-3) & -2(N-3)-3v & (N-3)(1-v) & \ddots & \vdots \\ \vdots & \ddots & \ddots & \ddots & \ddots & \vdots \\ 0 & \ddots & \ddots & \ddots & \ddots & 2(1-v) \\ -(1-v) & 0 & \cdots & \cdots & 1 & -2-(N-1)v \end{pmatrix}.$$

Therefore, the determinant can be calculated by applying LAPLACE expansion along the first column and evaluating the determinant of the remaining triangular matrix.

$$\begin{aligned} detP_1' &= (1-v) \begin{vmatrix} (N-1)(1-v) & 0 & \cdots & \cdots & \cdots & 0 \\ -2(N-2)-2v & (N-2)(1-v) & 0 & \ddots & \ddots & 0 \\ (N-3) & 2(N-3)-3v & (N-3)(1-v) & \ddots & \ddots & \vdots \\ 0 & \ddots & \ddots & \ddots & \ddots & \vdots \\ \vdots & \ddots & \ddots & \ddots & \ddots & 0 \\ 0 & \cdots & \ddots & \ddots & \ddots & 2(1-v) \end{vmatrix} \\ &= (1-v)(N-1)(1-v)(N-2)(1-v)(N-3)(1-v)...2(1-v) \\ &= (N-1)!(1-v)^{N-1}. \end{aligned} \tag{S6}$$

Using the calculated determinants from equations (S5) and (S6) allows the calculation of the absorption probability $\alpha^N(1, v)$ with equation (S4).

$$\alpha^N(1,v) = \frac{\det P_1'}{\det P'} = \frac{1}{P_{N-1}\left(\frac{v+1}{1-v}\right)}. \tag{S7}$$

Asymptotic absorption probabilities

Here, we derive the absorption probability in dependency of the risk coefficient γ as the system size N tends to infinity.
The second assumption on the parameter regime of the TGP model reads $(N\sqrt{v})^2 = \gamma$. Hence, $v = \frac{\gamma}{N^2}$. Substitution in equation (S7) yields

$$\alpha^N(\gamma) := \alpha^N(1,\gamma) = \frac{1}{P_{N-1}\left(\frac{v+1}{1-v}\right)} = \frac{1}{P_{N-1}\left(\frac{\frac{\gamma}{N^2}+1}{1-\frac{\gamma}{N^2}}\right)} = \frac{1}{P_{N-1}\left(\frac{N^2+\gamma}{N^2-\gamma}\right)}. \tag{S8}$$

One important property of the LEGENDRE polynomials is the integral representation (see [2])

$$P_N(x) = \frac{1}{\pi}\int_0^\pi \left[x + \sqrt{x^2-1}\cos\varphi\right]^N \mathrm{d}\varphi, \quad x \in \mathbb{R}\backslash\{-1,1\}.$$

This integral representation is used in order to calculate the limit for the denominator in (S8) as N goes to infinity.

$$\begin{aligned}
P_{N-1}\left(\frac{N^2+\gamma}{N^2-\gamma}\right) &= \frac{1}{\pi}\int_0^\pi \left[\frac{N^2+\gamma}{N^2-\gamma} + \sqrt{\left(\frac{N^2+\gamma}{N^2-\gamma}\right)^2 - 1}\cos\varphi\right]^{N-1} \mathrm{d}\varphi \\
&= \frac{1}{\pi}\int_0^\pi \left[\frac{N^2+\gamma}{N^2-\gamma} + \frac{\sqrt{(N^2+\gamma)^2-(N^2-\gamma)^2}\cos\varphi}{N^2-\gamma}\right]^{N-1} \mathrm{d}\varphi \\
&= \frac{1}{\pi}\int_0^\pi \left[\frac{N^2+\gamma+2N\sqrt{\gamma}\cos\varphi}{N^2-\gamma}\right]^{N-1} \mathrm{d}\varphi. \qquad \text{(S9)}
\end{aligned}$$

Therefore,

$$\begin{aligned}
\lim_{N\to\infty} P_{N-1}\left(\frac{N^2+\gamma}{N^2-\gamma}\right) &= \lim_{N\to\infty}\frac{1}{\pi}\int_0^\pi \left[\frac{N^2+\gamma+2N\sqrt{\gamma}\cos\varphi}{N^2-\gamma}\right]^{N-1} \mathrm{d}\varphi \\
&= \frac{1}{\pi}\int_0^\pi \lim_{N\to\infty}\left[\frac{N^2+\gamma+2N\sqrt{\gamma}\cos\varphi}{N^2-\gamma}\right]^{N-1} \mathrm{d}\varphi. \qquad \text{(S10)}
\end{aligned}$$

The exchange of the limit and the integral is justified by using LEBESGUE's dominated convergence theorem since there is an integrable majorant which can be derived as follows.

$$\begin{aligned}
\left|\frac{N^2+\gamma+2N\sqrt{\gamma}\cos\varphi}{N^2-\gamma}\right|^{N-1} &\leq \left|\frac{N^2+\gamma+2N\sqrt{\gamma}}{N^2-\gamma}\right|^{N-1} \\
&= \left|\frac{(N+\sqrt{\gamma})^2}{(N-\sqrt{\gamma})(N+\sqrt{\gamma})}\right|^{N-1} \\
&= \left|\frac{N+\sqrt{\gamma}}{N-\sqrt{\gamma}}\right|^{N-1} \\
&= \left|1+\frac{2\sqrt{\gamma}}{N-\sqrt{\gamma}})\right|^{N-1} \\
&= \left|\left[1+\frac{2\sqrt{\gamma}}{N-\sqrt{\gamma}}\right]^{N-\sqrt{\gamma}}\left[1+\frac{2\sqrt{\gamma}}{N-\sqrt{\gamma}}\right]^{\sqrt{\gamma}-1}\right| \\
&\leq \exp(2\sqrt{\gamma})\cdot 2
\end{aligned}$$

for N sufficiently large and since the sequence $\left(1+\frac{1}{N}\right)^N$ is monotonously increasing. The limit in (S10) can be calculated as follows.

$$\begin{aligned}\lim_{N\to\infty}\left[\frac{N^2+\gamma+2N\sqrt{\gamma}\cos\varphi}{N^2-\gamma}\right]^{N-1} &= \lim_{N\to\infty}\left[1+\frac{2\gamma+2N\sqrt{\gamma}\cos\varphi}{N^2-\gamma}\right]^{N-1}\\ &= \exp(2\sqrt{\gamma}\cos\varphi)\end{aligned}$$

since

$$\frac{2\gamma+2N\sqrt{\gamma}\cos\varphi}{N-\frac{\gamma}{N}} \to 2\sqrt{\gamma}\cos\varphi$$

and $a_N \to a$ implies $(1+\frac{a_N}{N})^N \to \exp(a)$. Hence, we obtain for (S10)

$$\begin{aligned}\frac{1}{\pi}\int_0^\pi \lim_{N\to\infty}\left[\frac{N^2+\gamma+2N\sqrt{\gamma}\cos\varphi}{N^2-\gamma}\right]^{N-1}\mathrm{d}\varphi &= \frac{1}{\pi}\int_0^\pi \exp(2\sqrt{\gamma}\cos\varphi)\mathrm{d}\varphi\\ &= I_0(2\sqrt{\gamma}),\end{aligned}$$

where I_n denotes the modified Bessel function of the first kind. This function can be expressed by the series

$$I_n(x) = \sum_{m=0}^{\infty}\frac{1}{m!\Gamma(m+n+1)}\left(\frac{x}{2}\right)^{2m+n} \tag{S11}$$

where $\Gamma(x)$ denotes the Gamma function, see [2] for details. Now, equation (S9) implies

$$\alpha(\gamma) := \lim_{N\to\infty}\alpha^N(1,\gamma) = \lim_{N\to\infty}\frac{1}{P_{N-1}\left(\frac{N^2+\gamma}{N^2-\gamma}\right)} = \frac{1}{I_0(2\sqrt{\gamma})}. \tag{S12}$$

A comparison between simulation results produced by sampling trajectories of the process $(X_t)_{t\geq 0}$, absorption probabilities obtained by using the exact formula (S7) and the asymptotic equation (S12) is given in Table S1.

Derivation of the regression function

The regression function $\beta_\gamma^N(\rho)$ can be estimated by a diffusion approximation of the TGP process. In order to achieve this, a derivation in [1] is used and extended. There, it is shown that it suffices to investigate a modified process $(Y_t)_{t\geq 0}$. This process is determined by the original rates given by (S1) with the following modification. The rate for a type-I mutation equals $u=0$ in $q(k,l)$ if $k>0$. Hence, type-I mutations are not allowed if type-I cells are already present in the system. The modification can be justified by the

assumption $u \ll \frac{1}{N}$ which allows to treat each mutant lineage independently. Note that this decomposition was already used in the calculation of the absorption probabilities. There, the state space has been reduced by state 0 since the occurrence of the successful mutant is assumed at the beginning. For the process $(Y_t)_{t\geq 0}$, this reduction of the state space is not sensible since we want to investigate the regression probability, i.e. the probability of reaching state 0. Hence, both modified processes only differ in the possibility of reaching state 0.

Under appropriate time and space scaling, $(Y_t)_{t\geq 0}$ can be asymptotically approximated for $N \to \infty$ by the WRIGHT-FISHER diffusion process Z_t on $[0,1]$. The details of this construction can be found in [1]. The important connection between the processes $(Y_t)_{t\geq 0}$ and $(Z_t)_{t\geq 0}$ is that $Z_t = 0$ implies $X_t = 0$.

Therefore, we approximate $\beta_\gamma^N(\rho) \approx \beta_\gamma(\rho)$ where $\beta_\gamma(\rho)$ is the probability that Z_t reaches 0 when starting in ρ,

$$\beta_\gamma(\rho) := \mathbb{P}(Z_t = 0 \text{ for some } t > 0 | Z_0 = \rho), \quad 0 \leq \rho \leq 1.$$

It holds that

$$\beta_\gamma(\rho) = c \sum_{k=1}^{\infty} \frac{\gamma^k}{k!(k-1)!}(1-\rho)^k, \quad 0 \leq \rho \leq 1, \tag{S13}$$

where the constant c is determined by the condition $\beta_\gamma(0) = 1$. See [1, Lemma 6.9] for details and a rigorous derivation of this approximation.

Here, we express the series representation (S13) in terms of BESSEL functions and derive the constant c as follows. In the first step, the indices of the sum are adjusted. In the second step, we used that $\Gamma(n) = (n-1)!$ for $n \in \mathbb{N}$.

$$\begin{aligned}
\sum_{k=1}^{\infty} \frac{\gamma^k}{k!(k-1)!}(1-\rho)^k &= \sum_{k=0}^{\infty} \frac{1}{(k+1)!k!}(\gamma(1-\rho))^{k+1} \\
&= \sum_{k=0}^{\infty} \frac{1}{k!\Gamma(k+2)}(\gamma(1-\rho))^{k+1} \\
&= \sum_{k=0}^{\infty} \frac{1}{k!\Gamma(k+2)}\left(\sqrt{\gamma(1-\rho)}\right)^{2k+2} \\
&= \sqrt{\gamma(1-\rho)} \sum_{k=0}^{\infty} \frac{1}{k!\Gamma(k+2)}\left(\sqrt{\gamma(1-\rho)}\right)^{2k+1} \\
&= \sqrt{\gamma(1-\rho)} I_1\left(2\sqrt{\gamma(1-\rho)}\right).
\end{aligned}$$

In the last step the definition of the modified BESSEL function of the first kind (S11) was utilized with $x = 2\sqrt{\gamma(1-\rho)}$ and $n = 1$. Hence, equation (S13) yields

$$\beta_\gamma(\rho) = c\sqrt{\gamma(1-\rho)} I_1\left(2\sqrt{\gamma(1-\rho)}\right).$$

Since $\beta_\gamma(0) = 1$, one can conclude that the constant c equals

$$c = \frac{1}{\sqrt{\gamma} I_1(2\sqrt{\gamma})}$$

and therefore

$$\beta_\gamma(\rho) = \frac{\sqrt{1-\rho} I_1\left(2\sqrt{\gamma(1-\rho)}\right)}{I_1(2\sqrt{\gamma})} \tag{S14}$$

for $0 \leq \rho \leq 1$.

Taylor expansion of the regression function

The Taylor expansion of the regression function is derived as follows. Since the regression function is defined for $0 \leq \rho \leq 1$, we expand it at $\rho_0 = 0.5$. Furthermore, an estimate for the remainder term is given.

The first order Taylor polynomial at x_0 of an two times differentiable function f is given by

$$T_1(x) = f(x_0) + f'(x_0)(x - x_0) + R_1(x),$$

where $R_1(x) = f(x) - T_1(x)$ denotes the remainder term. The Lagrange form of the remainder term is

$$R_1(x) = \frac{f''(\xi)}{2}(x - x_0)^2$$

for some real number ξ between x_0 and x.

We choose

$$f(\rho) = \beta_\gamma(\rho) = \frac{\sqrt{1-\rho} I_1\left(2\sqrt{\gamma(1-\rho)}\right)}{I_1(2\sqrt{\gamma})},$$

i.e. the regression function derived in equation (S14). The first two derivatives of f are given by

$$f'(\rho) = -\frac{I_1\left(2\sqrt{\gamma(1-\rho)}\right) + \sqrt{\gamma(1-\rho)}\left(I_0\left(2\sqrt{\gamma(1-\rho)}\right) + I_2\left(2\sqrt{\gamma(1-\rho)}\right)\right)}{2\sqrt{1-\rho} I_1(2\sqrt{\gamma})},$$

$$f''(\rho) = \gamma \frac{I_1\left(2\sqrt{\gamma(1-\rho)}\right)}{I_1(2\sqrt{\gamma})\sqrt{(1-\rho)}}.$$

Here, it was used that

$$\frac{d}{dx} I_0(x) = I_1(x) \quad \text{and}$$

$$\frac{d}{dx} I_m(x) = \frac{I_{m-1}(x) + I_{m+1}(x)}{2} = I_{m-1}(x) - \frac{m I_m(x)}{x}, \quad m \in \mathbb{N},$$

see [2]. Therefore, for $\rho_0 = 0.5$, it holds that

$$T_1(\rho) = \frac{I_1\left(\sqrt{2\gamma}\right)}{\sqrt{2}I_1\left(2\sqrt{\gamma}\right)} - \frac{\sqrt{2}I_1\left(\sqrt{2\gamma}\right) + \sqrt{\gamma}\left(I_0\left(\sqrt{2\gamma}\right) + I_2\left(\sqrt{2\gamma}\right)\right)}{2I_1\left(2\sqrt{\gamma}\right)}(\rho - 0.5). \tag{S15}$$

The remainder term for $0 \leq \rho \leq 1$ can be estimated in the following way. First, note that for $\rho \in [0, 1]$ it holds that

$$f''(\rho) = \gamma \frac{I_1\left(2\sqrt{\gamma(1-\rho)}\right)}{I_1(2\sqrt{\gamma})\sqrt{(1-\rho)}} \leq \gamma \frac{I_1\left(2\sqrt{\gamma(1-\rho)}\right)}{I_1(2\sqrt{\gamma})} \leq \gamma$$

since $I_1(x)$ is monotonously increasing. Therefore, $\max_{\rho \in [0,1]} f''(\rho) = f''(0) = \gamma$ and

$$\begin{aligned} |R_1(\rho)| &= \left| \frac{f''(\xi)}{2}(\rho - 0.5)^2 \right| \\ &= \left| \frac{\gamma I_1\left(2\sqrt{\gamma}\sqrt{1-\xi}\right)}{I_1(2\sqrt{\gamma})\sqrt{1-\xi}} \frac{(\rho - 0.5)^2}{2} \right| \\ &\leq \gamma \frac{(1-0.5)^2}{2} \\ &= \frac{\gamma}{8}. \end{aligned} \tag{S16}$$

References

1. Durrett R, Schmidt D, Schweinsberg J. A waiting time problem arising from the study of multi-stage carcinogenesis. Ann Appl Proba. 2009;19(2):676–718.

2. Abramowitz M, Stegun IA. Handbook of Mathematical Functions - with Formulas, Graphs, and Mathematical Tables.
