# Supplementary figures and images for: Model-Based Evaluation of Spontaneous Tumor Regression in Pilocytic Astrocytoma
Source: PLoS Comput Biol. 2015 Dec 10;11(12):e1004662. doi: 10.1371/journal.pcbi.1004662 (PMC4675550; doi:10.1371/journal.pcbi.1004662)

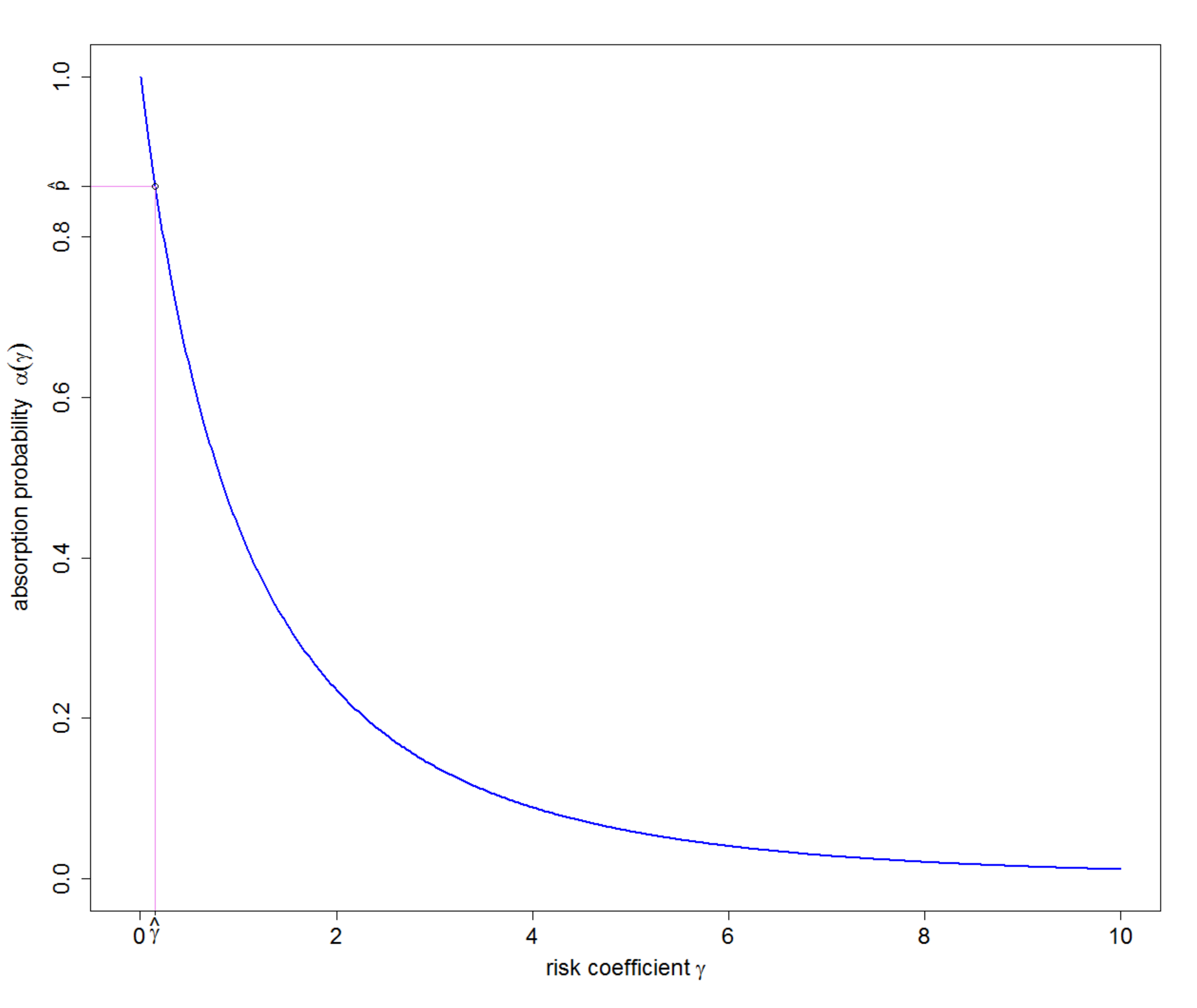

Supplement: S1 Fig — The asymptotic absorption probability α(γ) in state N is strictly monotonically decreasing. The clinically observed fraction of PA-I cases is estimated as p^=0.8634 and the corresponding risk coefficient is γ^=0.152. (TIF) [file pcbi.1004662.s002.tif]
