# Supplementary material for: Model-Based Evaluation of Spontaneous Tumor Regression in Pilocytic Astrocytoma
Source: PLoS Comput Biol. 2015 Dec 10;11(12):e1004662. doi: 10.1371/journal.pcbi.1004662 (PMC4675550; doi:10.1371/journal.pcbi.1004662)
Supplement: S1 Table — This table contains the results of our literature research about volumetric data of residual cerebellar PA and the corresponding patient outcome. (PDF) [file pcbi.1004662.s003.pdf]

residual tumor size (cm^3)	outcome	reference
0.1	tumor regression	[2]
0.1	tumor regression	[1]
2	tumor regression	[2]
2	tumor regression	[2]
2	tumor regression	[2]
2	tumor regression	[2]
2.5	tumor regression	[1]
6	tumor regression or stable	[2]
8.8	tumor regression or stable	[2]
10	tumor growth	[2]
13.6	tumor growth	[1]
16.8	tumor growth	[2]
43.2	tumor growth	[2]

Table S1. Residual cerebellar postoperative PA volume and outcome. This table contains the results of our literature research regarding volumetric data of residual cerebellar PA and the corresponding patient outcome.

References

1. Smoots D, Geyer J, Lieberman D, Berger M. Predicting disease progression in childhood cerebellar astrocytoma. Childs Nerv Syst. 1998;14:636–648.

2. Gunny RS, Hayward RD, Phipps KP, Harding BN, Saunders DE. Spontaneous regression of residual low-grade cerebellar pilocytic astrocytomas in children. Pediatr Radiol. 2005;35(11):1086–1091.
