# Supplementary material for: Model-Based Evaluation of Spontaneous Tumor Regression in Pilocytic Astrocytoma
Source: PLoS Comput Biol. 2015 Dec 10;11(12):e1004662. doi: 10.1371/journal.pcbi.1004662 (PMC4675550; doi:10.1371/journal.pcbi.1004662)
Supplement: S2 Table — This table shows the exact absorption probabilities α N(γ) in state N compared to the results from 10000 simulations of trajectories of the TGP process (X t)t ≥ 0 and the asymptotic absorption probabilities α(γ). The asymptotic values are in good accordance with both, the exact theoretical values and the simulation results. Furthermore, the asymptotic result α(γ) is a good approximation even for small N. (PDF) [file pcbi.1004662.s004.pdf]

N	u	v	γ	$\alpha^N(\gamma)$	$\alpha(\gamma)$	simulated fraction of absorption
10	10^{-4}	2500^{-1}	0.04	0.9649	0.96117	0.9632
100	10^{-4}	2500^{-1}	4	0.09	0.0885	0.0921
10	10^{-4}	10000^{-1}	0.01	0.9911	0.9901	0.9912
100	10^{-4}	10000^{-1}	1	0.4417	0.4387	0.4419
10	10^{-4}	1.52×10^{-3}	0.152	0.8759	0.8636	0.882
100	10^{-4}	1.52×10^{-5}	0.152	0.8649	0.8636	0.8665

Table S2. Comparison of theoretical absorption probabilities and simulation results. This table shows the exact absorption probabilities $\alpha^N(\gamma)$ in state N in comparison with the results from 10000 simulations of trajectories of the process $(X_t)_{t\geq 0}$ and the asymptotic absorption probabilities $\alpha(\gamma)$. One can see that the asymptotic values are in good accordance with both, the exact theoretical values and the simulation results. Furthermore, the asymptotic result $\alpha(\gamma)$ is a good approximation even for small N.
